# Metrnl inhibits choroidal neovascularization by attenuating the choroidal inflammation via inactivating the UCHL-1/NF-κB signaling pathway

**DOI:** 10.3389/fimmu.2024.1379586

**Published:** 2024-04-30

**Authors:** Lanyue Zhang, Youjian Li, Zhengyu Wu, Qiang Shen, Chunqin Zeng, Han Liu, Xuedong Zhang, Jiaxing Yang, Qiaoling Liu, Dianyong Tang, Kepeng Ou, Yanhong Fang

**Affiliations:** ^1^ Department of Ophthalmology, Chongqing University Jiangjin Hospital, Chongqing, China; ^2^ College of Pharmacy, National & Local Joint Engineering Research Center of Targeted and Innovative Therapeutics, International Academy of Targeted Therapeutics and Innovation (IATTI), Chongqing University of Arts and Sciences, Chongqing, China; ^3^ Chongqing Key Laboratory of Ophthalmology, Chongqing Eye Institute, Chongqing Branch (Municipality Division) of National Clinical Research Center for Ocular Diseases, The First Affiliated Hospital of Chongqing Medical University, Chongqing, China

**Keywords:** Metrnl, choroidal neovascularization, macrophage, NF-κB signaling pathway, UCHL-1

## Abstract

**Objective:**

Choroidal neovascularization (CNV) represents the predominant form of advanced wet Age-related Macular Degeneration (wAMD). Macrophages play a pivotal role in the pathological progression of CNV. Meteorin-like (Metrnl), a novel cytokine known for its anti-inflammatory properties in macrophages, is the focus of our investigation into its mechanism of action and its potential to impede CNV progression.

**Methods:**

Cell viability was evaluated through CCK-8 and EdU assays following Metrnl treatment. Expression levels of inflammatory cytokines and proteins were assessed using quantitative reverse‐transcription polymerase chain reaction(qRT‐PCR), enzyme-linked immunosorbent assay (ELISA), and western blot techniques. Protein-protein interactions were identified through protein mass spectrometry and co-immunoprecipitation (Co‐IP). Additionally, *in vivo* and *in vitro* neovascularization models were employed to evaluate angiogenesis.

**Results:**

Our results revealed downregulated Metrnl levels in the choroid-sclera complex of CNV mice, the aqueous humor of wAMD patients, and activated macrophages. Metrnl overexpression demonstrated a reduction in pro-inflammatory cytokine production, influenced endothelial cell function, and suppressed angiogenesis in choroid explants and CNV models. Through protein mass spectrometry and Co‐IP, we confirmed Metrnl binds to UCHL-1 to modulate the NF-κB signaling pathway. This interaction inhibited the transcription and expression of pro-inflammatory cytokines, ultimately suppressing angiogenesis.

**Conclusion:**

In summary, our findings indicate that Metrnl down-regulates macrophage pro-inflammatory cytokine secretion via the UCHL-1/NF-κB signaling pathway. This mechanism alleviates the inflammatory microenvironment and effectively inhibits choroidal neovascularization.

## Introduction

Age-related macular degeneration (AMD) is a progressive and degenerative disease that leads to irreversible visual damage in the elderly. As the population ages, the number of AMD patients is expected to increase to 288 million by 2040, particularly in Asia, where the population may grow to 113 million (1). Choroidal neovascularization (CNV) is common in advanced stages of wet AMD (wAMD), characterized by blood or serum leakage. Following damage to the Bruch membrane, choroidal capillaries extend into the retinal pigment epithelium (RPE), resulting in neovascularization and severe visual loss (2). Poor neovascularization structure and function result in various outcomes such as exudation, hemorrhage, and scarring (2). The pathogenesis of CNV remains unclear, despite our understanding that inflammation contributes to early CNV (3). As macrophages and other inflammatory cells infiltrate, numerous inflammatory cytokines are released at the lesion, amplifying vascular inflammation and intensifying angiogenesis (4).

Under physiological conditions, tissue-resident macrophages, a type of innate immune mononuclear phagocyte cells, reside in the choroid. Pathologically, macrophages recruited from the blood become activated alongside the original macrophages, promoting the onset of CNV (5). Inflammatory cytokines secreted by macrophages directly influence CNV progression; for instance, IL-1β activates innate immunity related to inflammation, and TNF-α up-regulates VEGF production (5). Additionally, activated macrophages recruit other cell types, such as mesenchymal cells, contributing to the template for CNV (6).

Meteorin-like (Metrnl) is a novel cytokine belonging to an evolutionarily conserved two-member protein family, together with Metrn (7). Recent reports have linked Metrnl to energy metabolism, insulin resistance, and immune regulation, generating increasing interest in its anti-inflammatory effects. Studies have shown that Metrnl, secreted by macrophages, could regulate the production of several cytokines and chemokines in macrophages (8, 9). It is now understood that Metrnl not only reduces the expression of cytokines such as IL-6 and IL-1β (8, 9), but also regulates macrophage infiltration in pathological conditions (10, 11). Lack of Metrnl inhibits anti-inflammatory effects and leads to the failure of class II MHC expression in macrophages, exacerbating inflammation (12).

Herein, we explored whether Metrnl could modulate the inflammatory response of macrophages, impacting endothelial cell function and the development of CNV. These findings provide new insights into treating CNV with Metrnl, providing a potential therapeutic strategy to slow its progression.

## Methods

### Clinical sample

This research encompassed both newly diagnosed wAMD patients and control patients with simple cataracts, selected by two experts based on fundus fluorescein angiography (FFA) and indocyanine green angiography (ICGA) assessments. Aqueous humor was collected from wAMD patients during their first intravitreal injection, while controls underwent cataract surgery. Following centrifugation at 3000 rpm for 5 minutes, samples were stored at -80°C before analysis. All participants provided written, informed consent, and the study received approval from the institute’s ethics committee at Chongqing University Jiangjin Hospital (approval reference number: KY2023006).

### Animal husbandry

Adult C57BL/6 mice (4-8 weeks old) were housed at Chongqing University of Arts and Sciences, National & Local Joint Engineering Research Center of Targeted and Innovative Therapeutics, Chongqing, China. Ethical approval for all animal experiments was obtained from the Experimental Animal Welfare Ethics Committee of Chongqing University of Arts and Sciences (approval reference number: CQWLDF202304), adhering to the declaration on the use of animals in ophthalmic and vision research by the Association for Research in Vision and Ophthalmology (ARVO).

### Mice CNV model

After dilating pupils with compound tropicamide eye drops (Shenyang Sinqi Pharmaceutical Co., Ltd.), C57BL/6 mice underwent laser photocoagulation. Laser spots were induced in each of the four quadrants using an OculightSlx Krypton Red Laser system (power 200 mW, duration 75 ms, spot size 75 μm). One day after laser treatment, 2 μl of Metrnl (100ng/ml) or PBS was injected into the vitreous. Mice were euthanized one week later for immunohistochemistry assays and qRT‐PCR.

### Cell culture

Primary human umbilical vein endothelial cells (HUVECs) were cultured in endothelial cell growth medium (ECM, Promocell). Human embryonic kidney 293T (HEK293T) and mouse macrophages (RAW264.7 cells) cultures were performed in DMEM (purchased from Procell Company, Wuhan, China) containing 10% FBS. Human macrophages (THP-1 cells) were cultured in RPMI-1640 (Life Technologies) with 10% FBS (Biological Industries). These cells were placed in 37°C, 5% CO_2_ incubator.

All of cell lines present in this study were obtained from Zhejiang Meisen Cell Technology Co., Ltd.

### Cell viability and proliferation assay

Cell viability was determined using the CCK-8 assay (Beyotime). In brief, before detecting the optical density (OD) at 450 nm using a microplate reader, each culture well was added with 10μl of CCK-8 solution and was incubated for 2h at 37°C with 5% CO_2_.

EdU cell proliferation staining was performed using an EdU kit (Beyotime). Briefly, cells (8 × 10^3^ cells/well) were cultured with/without Metrnl (100nM) on round coverslips in 24-well plates for 24 h. After fixed with 4% paraformaldehyde and permeated with 0.3% Triton X-100 for 15 min, cells were incubated with EdU for 2h. They were treated with the Click Reaction Mixture for 30 min at room temperature in a dark place before being counterstained with Hoechst 33342 for 10 minutes.

### ELISA

The levels of Metrnl, IL-1β, IL-6, and TNF-α in clinical aqueous humor, choroid of mice, and the media of RAW264.7 cell cultures were measured using ELISA kits (Abclonal Technology, Wuhan, China/MeiKe, Jiangsu, China) following the provided protocols.

### Western blot

Cells were lysed using a nuclear and cytoplasmic protein extraction kit (Beyotime, Shanghai, China). Protein concentration was determined with a BCA kit (Beyotime, Shanghai, China). Subsequently, the protein samples underwent separation through SDS-PAGE gel electrophoresis and were then transferred to PVDF membranes. After blocking with 5% skimmed milk for 1 hour, the membranes were incubated overnight at 4°C with primary antibodies: Anti-Metrnl (ABclonal, USA; 1:1000), anti-IκBα (ABclonal, USA; 1:1000), anti-NF‐κB (ABclonal, USA; 1:1000), PCNA (ABclonal, USA; 1:1000), and β-tubulin (Proteintech, USA; 1:1000). They were then treated for 1 hour at room temperature with HRP-conjugated secondary antibodies. Visualization of the bands was achieved using ECL reagents (ThermoFisher, MA, USA).

### Co-immunoprecipitation

Cells were lysed with NP40 solution for 30 minutes on ice. Following centrifugation, the supernatant was collected in the EP tube. *In vitro* CO-IP assay, magnetic beads were incubated with the rest of supernatants for overnight at 4°C. *In vivo* CO-IP assay, after incubation with antibodies for 6 hours, the magnetic beads were incubated overnight with the collected supernatant. Then, these magnetic beads were separated from the mixture and resuspended in 80μl NP-40 lysis buffer, which were mixed with 20μl of SDS-loading buffer to boil for 10 minutes. Finally, samples were collected for Western blot.

### Quantitative reverse‐transcription polymerase chain reaction analysis

The total RNA was extracted from RAW264.7and THP-1cells with RNAfast200 (Fastagen, Shanghai, China). The purity and amounts of RNA were estimated by spectrophotometer. Then, cDNA was gained though reverse transcription process with HiScript IV RT SuperMix for qPCR (Vazyme, Nanjing, China). SYBR Green Real-time PCR Master Mix (Vazyme, Nanjing, China) was used for qRT-PCR. The primer sequences were designed from the PrimerBank database (**Supplementary Table 1**).

### HUVEC tube formation and migration assay

Initially, three kinds of conditioned medium were collected from RAW264.7 cells. The first was normal conditioned medium (N-CM), the second was conditioned medium stimulated by LPS (L-CM), and the last was conditioned medium from the cells overexpressing Metrnl, meanwhile stimulated by LPS (M-CM). In 96-well plates, 50ul of Matrigel (BD Biosciences) was applied per well and placed at 37°C for 30 min to solidify. Then P5 HUVECs (1.5× 10^4^) cells were seeded on top and categorized into three groups. Each group received 50 μl of ECM and 50 μl of the previously mentioned conditioned medium. Photographs were taken at 4h, 12h, and 24h intervals.

HUVECs were seeded in 6-well plates and incubated until reaching full confluence. After overnight incubation in serum-free medium, cells were scratched to create an artificial wound using a 200 μL pipette tip. The scratched cells were washed off with PBS, and each well received 1ml of DMEM and 1ml of the aforementioned conditioned medium, incubated at 37°C, 5% CO_2_. Scratch areas were photographed at 0, 12, and 24 hours. The migration rate at 24 hours was calculated as follows: Migration rate at 24 hours = (width at 0 hours - width at 24-hour time point)/width at 0 hours × 100%.

All data were quantified using ImageJ 1.46r (National Institutes of Health, USA).

### Transwell migration assay

Transwell chambers were placed into 24-well plates. A total of 4 × 10^4^ HUVECs were resuspended in 200 μl of serum-free DMEM, evenly distributed in the upper chamber. The lower chambers were separately supplemented with 400 μl of DMEM and 400 μl of the aforementioned conditioned medium. After 12 hours, the membranes in the upper chambers were fixed for 30 minutes in 4% paraformaldehyde and stained for 20 minutes in Crystal Violet Staining Solution at room temperature. Using a x20 microscope, five fields of view were photographed for each membrane.

### Choroidal explant assay

As described in previous experiments (13), we extracted the choroid-sclera complex from 6-8 week old mice. The complex was subsequently sliced into 1-mm pieces and embedded into Matrigel (BD Biosciences). Once solidified, the pieces were treated with 200 μl of various conditioned medium and 200 μl of DMEM. After 10 days, photographs of choroidal explants were captured, and the sprouting areas were quantified using ImageJ (National Institutes of Health).

### Vitro vascular permeability model

Sterile coverslips were placed into a 24-well plate and incubated with poly-L-Lysine for 20 minutes. Following the protocols from Millipore, USA, coverslips were sequentially treated with glutaraldehyde, biotinylated-gelatin, and growth media. HUVECs were seeded on the coverslips and cultured until a confluent monolayer was achieved in a 37°C, 5% CO_2_ incubator. After an overnight incubation in serum-free medium, various supernatants were added to the plates as a stimulant for 12 hours. Before fixing cells with 3.7% formaldehyde, the culture media was removed, and cells were incubated with fluorescein-streptavidin for 5 minutes. Subsequently, each well was supplemented with anti-VE cadherin antibody overnight at 4°C and anti-Mouse IgG, Cy3 conjugate at room temperature for 1 hour. Following a brief washing, the coverslips were inverted onto a glass slide with slide mounting media. Finally, images were captured using fluorescence microscopy.

### Transfection

Serum-free DMEM was prepared and supplemented with HighGene plus Transfection reagent (ABclonal). Plasmids expressing genes such as Metrnl, UCHL-1, and others were added to the mixture. After thorough mixing, the mixture was transfected into HEK293T or THP-1 cells for 24 hours. When transfecting plasmids into RAW264.7 cells, the mixture was prepared in a similar manner and incubated for 24 hours. SiRNA targeting Metrnl and UCHL-1 were transfected using a comparable approach. All steps were carried out in accordance with the provided protocols.

### Statistical analysis

GraphPad Prism 9 was used for statistical analysis. The unpaired Student’s t test was applied to compare two separate experimental groups. The Analysis of Variance (ANOVA) test was utilized for nonparametric analysis of multiple comparisons. A P-value<0.05 was statistically significant.

## Results

### The expression of Metrnl was decreased in the CNV pathology samples and models

**Figure 1:**
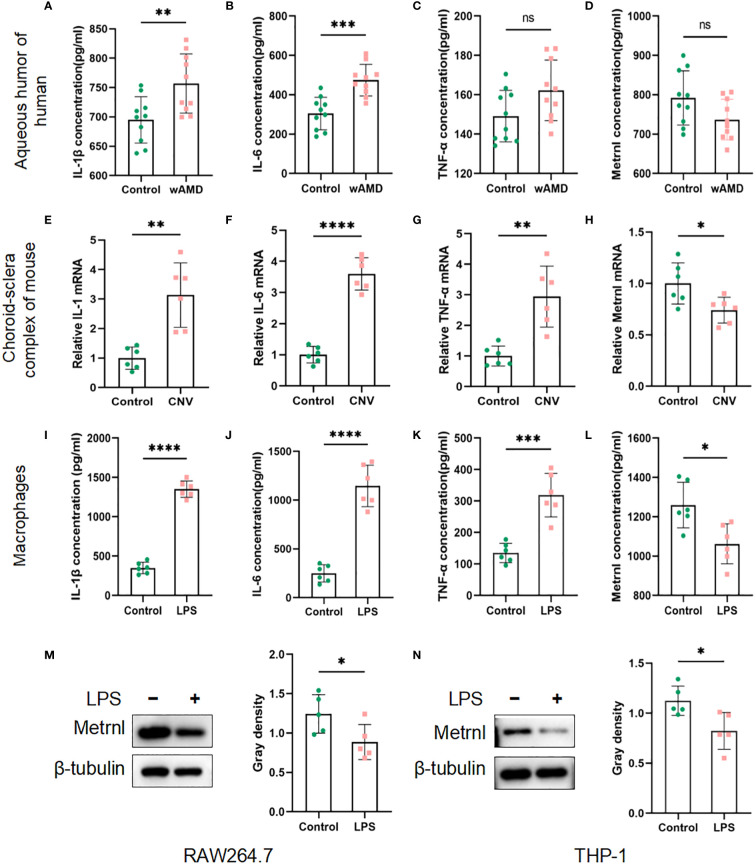
Concentrations of IL-1 β, IL-6, TNF-α, and Metrnl were assessed using ELISA kits, qRT-PCR and Western blot. **(A–D)** Expression levels of IL-1 β, IL-6, TNF-α, and Metrnl in human aqueous humor. **(E–H)** mRNA levels of IL-1, IL-6, TNF-α, and Met rnl in mouse choroid. **(I–L)** Expression of IL-1 β, IL-6, TNF-α , and Metrnl in conditioned medium from macrophages. **(M, N)** The protein level of Metrnl in LPS-treated macrophages. Data are presented as means ± SD, from 3 independent experiments. (ns >.05, , * P < .05; **P < .01, ***P < .001, and ****P < .0001).

The expression levels of inflammatory cytokines (IL-1β, IL-6, and TNF-α) and Metrnl were initially assessed using ELISA. In the samples of aqueous humor, IL-1β and IL-6 exhibited a significant increase compared to the control group, while there was no significant difference in TNF-α expression (**Figures 1A–C**). The concentration of Metrnl showed a decreasing trend in wAMD patients’ aqueous humor, although this was not statistically significant (**Figure 1D**). A similar trend was observed in the expression of IL-1β, IL-6, and TNF-α in LPS-induced macrophages (RAW 264.7 cells) and the choroid-sclera complex of CNV mice (**Figures 1E–G, I–K**). Consistent with wAMD patients’ aqueous humor, the expression of Metrnl was suppressed (**Figures 1H, L**). Furthermore, Metrnl was evaluated by western blot, revealing a decrease in the protein level of Metrnl in LPS-treated macrophages (**Figures 1M, N**). These findings suggest that both inflammatory cytokines and Metrnl play crucial roles in the occurrence and development of CNV.

### Metrnl suppressed macrophage activation

**Figure 2:**
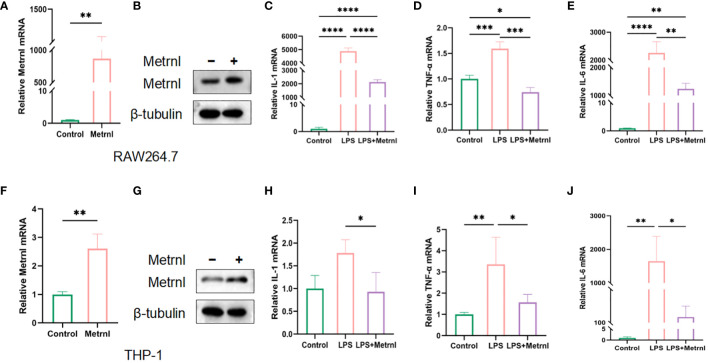
Metrnl affects the expression of inflammatory factors in LPS-induced macrophages. The efficiency of Metrnl transfection was tested by qRT-PCR and Western Blot in RAW264.7 cells **(A, B)** and THP-1 cells **(C, D)**. Gene expression of pro-inflammatory factors IL-1β, IL-6, and TNF-α among control group(Control), LPS-induced group(LPS), and Metrnl overexpression + LPS group(LPS+METRNL) in RAW264.7 cells **(C–E)** and THP-1 cells **(H–J)**. Data are presented as the mean ± SD of the relative values vs control, from 3 independent experiments. (*P <.05, **P <.01, ***P <.001, and ****P <.0001).

To investigate the impact of Metrnl on regulating macrophage activation, we initially examined the growth and proliferative ability of macrophages after the exogenous administration of Metrnl. The EdU assay revealed that increasing the expression of Metrnl did not affect the viability and proliferative ability of macrophages (**Supplementary Figures 1D, E**). We further investigated the function of Metrnl in the production of pro-inflammatory cytokines. Firstly, we validated the efficiency of overexpression of Metrnl in macrophages (**Figures 2A, B, F, G**). Subsequently, qRT-PCR was applied to assess the effects in different treated groups. RAW264.7 cells and THP-1 cells were treated with LPS to elevate mRNA levels of IL-1β, TNF-α, and IL-6, while overexpression of Metrnl significantly reduced them (**Figures 2C–E, H–J**). These results indicate that Metrnl can modulate pro-inflammatory cytokines in macrophages.

### Metrnl restored endothelial function stimulated by inflammatory cytokines

**Figure 3:**
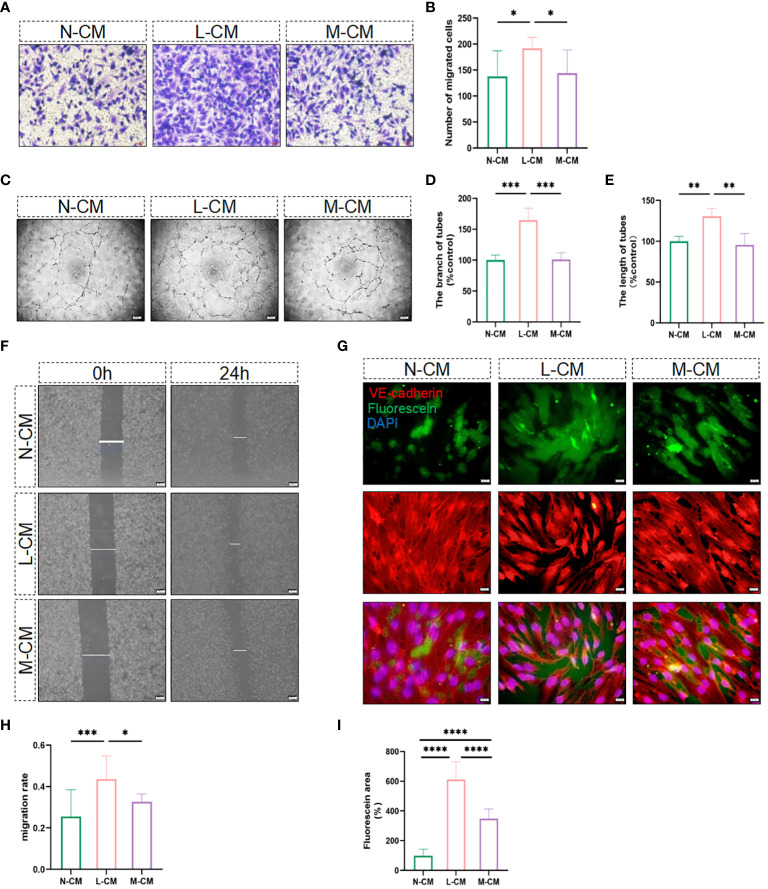
Metrnl impacts the function of endothelial cells. **(A, B, F, H)** Migratory capacity of HUVECs was performed by trans-well assay (Scale bar, 50 µm) and the gap closure migration assay (Scale bar, 200 µm). **(C–E)** N-CM group, L-CM group, and M-CM group regulate endothelial cell tube formation (Scale bar, 200 µm). **(G, I)** Immunostaining of HUVECs for fluorescein-streptavidin to detect permeable areas and VE-Cadherin to visualize cell-cell contact (Scale bar, 100 µm). Data are expressed as means ± SD, from 3 independent experiments. (*P <.05, **P <.01, ***P <.001, and ****P <.0001).

It is well known that activated macrophages play an essential role in regulating the process of angiogenesis. In the next step, we explored the role of Metrnl in regulating angiogenesis in activated macrophages. CCK-8 and EdU assays were employed to assess the influence of exogenous Metrnl on HUVECs, and the data illustrated that Metrnl did not impact the growth and proliferative ability of HUVECs (**Supplementary Figures 1A–C**). We then used conditioned medium from macrophages to assess the migratory ability of endothelial cells through migration assays (**Figures 3F, H**) and transwell experiments (**Figures 3A, B**). The results demonstrated that Metrnl significantly inhibited inflammation-induced endothelial cell migration. Furthermore, we explored whether Metrnl could influence the angiogenesis of HUVECs (**Figures 3C–E**). As expected, the L-CM group promoted tube formation, and the M-CM group could attenuate this effect. Additionally, it is well-established that the integrity and function of blood vessels are crucial in the process of CNV, where inflammatory cytokines significantly affect vascular permeability. We further employed an *in vitro* vascular permeability image model to assess the cell–cell adhesion of HUVECs. The permeability among HUVECs stimulated by L-CM was significantly worse than that in N-CM, and M-CM reversed the L-CM effect (**Figures 3G, I**). Therefore, the results suggest that Metrnl could reduce the permeability of neovascularization.

### Metrnl restrained angiogenesis through stabilizing macrophages ex vivo and *in vivo*

**Figure 4:**
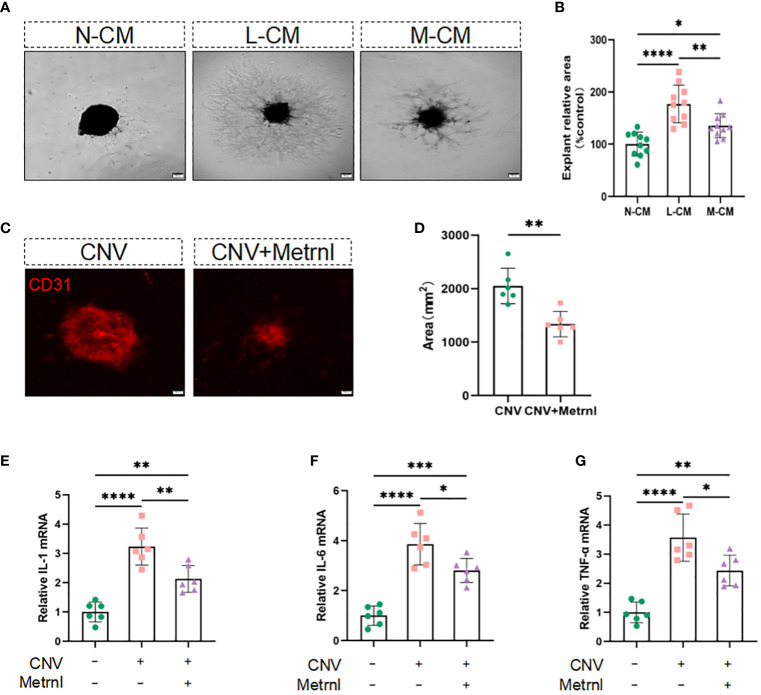
Metrnl suppresses angiogenesis through anti-inflammatory effects *in vitro* and *in vivo*. **(A, B)** Phase contrast photos of mouse choroidal sprouts were taken after 10 days culture with N-CM group, L-CM group, and M-CM group. **(C, D)** Immunostaining of choroidal neovascularization in mice was used to detect fluorescence area. **(E–G)** The mRNA levels of IL-1 β, IL-6, and TNF-α in choroid tissue from CNV mice. Data are expressed as means ± SD, from 3 independent experiments, scale bar, 200 μm. (*P < .05, **P < .01, ***P < .001, and ****P < .0001)

Considering that Metrnl affects endothelial cell function by attenuating LPS-mediated macrophage inflammatory responses, we sought to investigate its effect on angiogenesis in isolated choroidal explants. The results showed that the sprouting area of choroidal explants was increased in the L-CM group, which was mitigated in the M-CM group (**Figures 4A, B**). Inhibiting the inflammatory response is indeed beneficial for suppressing the process of CNV. Exogenous Metrnl was given in CNV model mice by intravitreal injection to assess the effect of Metrnl *in vivo*. Compared with the control group, Metrnl significantly suppressed the area of CNV (**Figures 4C, D**). Moreover, Metrnl reduced the levels of IL-1β, IL-6, and TNF-α in choroid tissue from CNV mice (**Figures 4E–G**). The results imply that Metrnl suppresses the process of CNV through anti-inflammatory effects.

### Metrnl directly interacts with UCHL-1 alleviates inflammatory response in macrophages

**Figure 5:**
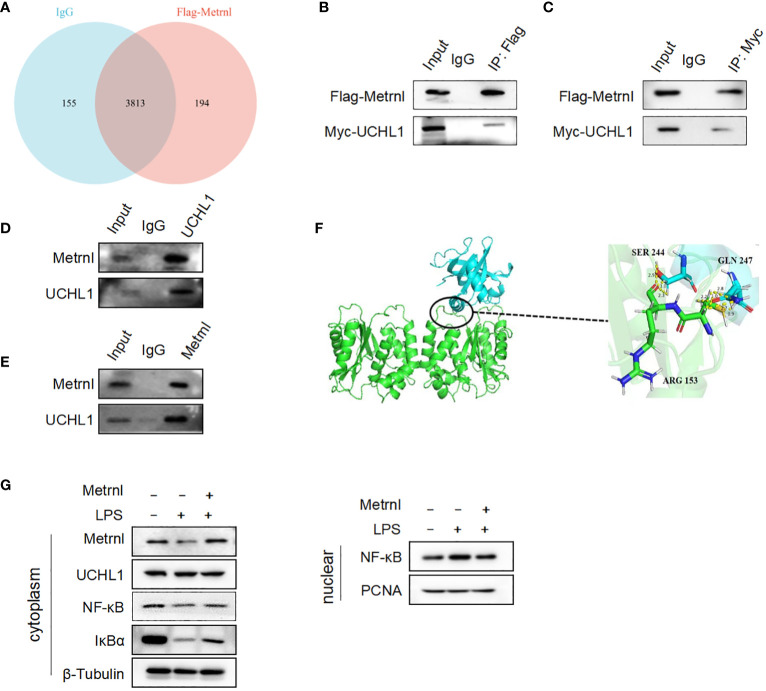
Metrnl directly interacts with UCHL-1 alleviating inflammatory response in macrophages. **(A)** Potential Metrnl-binding proteins were identified by mass spectrometry. **(B, C)** In HEK293T cell line, after co-transfection of Myc-UCHL-1 and Flag-Metrnl, IP assays were performed to enrich Myc-UCHL-1 and Flag-Metrnl. **(D, E)** In RAW264.7 cells, protein A/G after incubated with Metrnl or UCHL1 were incubated with extracted proteins overnight. Western blot detected enriched endogenous UCHL1 or Metrnl proteins **(F)** According to molecular docking technique, the binding site of UCHL-1 and Metrnl possible was serine 244, glutamine 247, or arginine 153. **(G)** Metrnl regulates NF-κB pathway through UCHL-1 in LPS-induced macrophages.

To assess the mechanism behind the regulatory effect of Metrnl on the secretion of pro-inflammatory factors in macrophages, mass spectrometry was applied to identify potential Metrnl-binding proteins. A total of 194 proteins were found to specifically bind to Metrnl (**Figure 5A**). After analyzing the abundance of these proteins, we identified highly enriched proteins potentially interacting with Metrnl and involved in inflammatory pathways. Next, these potential proteins were confirmed to interact with Metrnl through Co-IP assays. We constructed overexpression plasmids Myc-UCHL-1, Myc-CPT1B, and Myc-OTUD6B. Then, these plasmids were co-transfected with Flag-Metrnl in HEK293T cell lines, respectively. The Co-IP assay validated that Metrnl interacts with UCHL-1 both *in vitro* and *in vivo* (**Figures 5B–E**; **Supplementary Figures 2A, B**). Additionally, we predicted two potential interacting residues between Metrnl and UCHL-1 using molecular docking techniques. The molecular docking results showed that UCHL-1 and Metrnl could interact with each other, with putative binding sites being serine 244, glutamine 247, or arginine 153 (**Figure 5F**). Research evidence has shown that UCHL-1 is involved in the regulation of inflammation through activating/inactivating the NF-κB signaling pathway. We assessed the impact of Metrnl on the regulation of the UCHL-1/NF-κB pathway in macrophages (**Figure 5G**). The results showed that Metrnl did not regulate UCHL-1 expression but affected the downstream NF-κB signaling pathway of UCHL-1. NF-κB nuclear translocation was increased in LPS-induced macrophages; nevertheless, NF-κB nuclear translocation was inhibited with Metrnl overexpression. These results suggest that Metrnl may be involved in the NF-κB signaling pathway by binding and regulating UCHL-1 activity.

### Metrnl inhibited the activation of NF-κB signaling pathway dependent on UCHL-1

**Figure 6:**
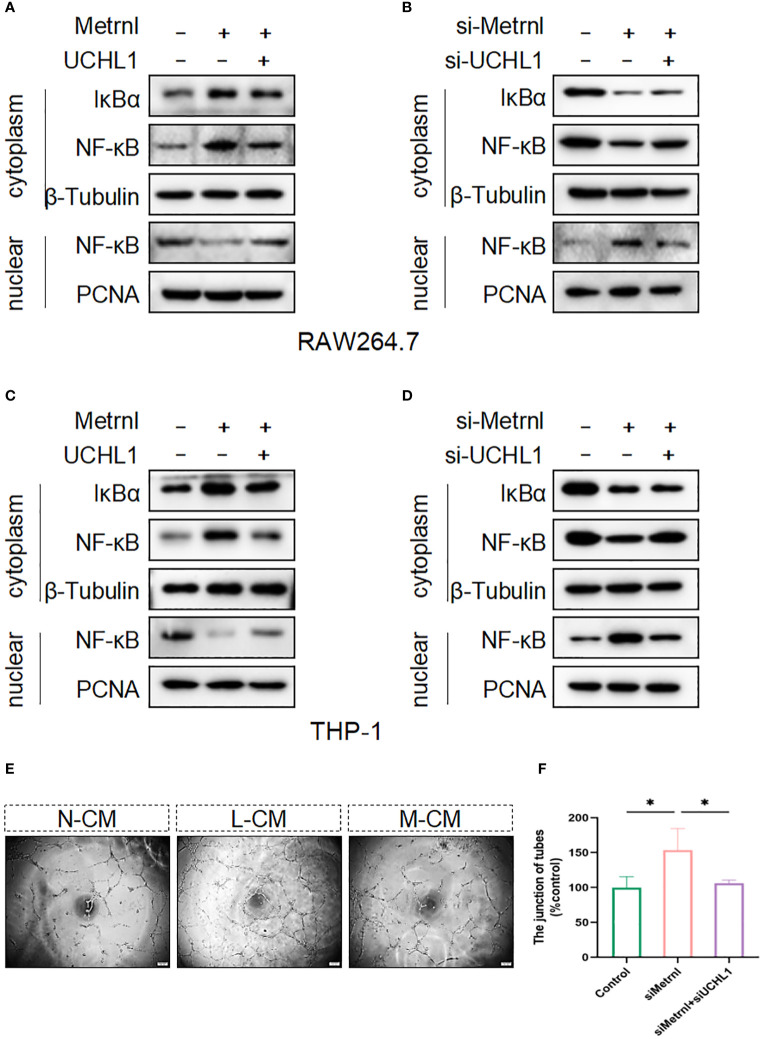
Metrnl regulates the activation of the NF-κB signaling pathway depending on UCHL-1 in human and mouse macrophages. **(A, C)** Plasmids were transfected into RAW264.7 cells and THP-1 cells to overexpress Metrnl and UCHL-1 for 24h. **(B, D)** RAW264.7 cells and THP‐1 cells were treated with UCHL-1 siRNA (100 nmol/L) and Metrnl siRNA (100 nmol/L). Then, nuclear and cytoplasmic proteins were extracted, and IκBα and NF‐κB p65 were assessed by western blot. The amounts of cytoplasmic and nuclear proteins were normalized to β‐tubulin and PCNA levels, respectively. **(E, F)** Conditioned media from the control group, si-Metrnl group, and si-Metrnl + si-UCHL-1 group treat endothelial cells to assess tube formation. Data are expressed as means ± SD of relative values vs. control from 3 independent experiments. (* P < 0.05; statistical analysis was performed using one-way ANOVA with Dunn’s test for multiple comparisons).

To validate that Metrnl relies on UCHL-1 to regulate the activation of inflammatory signaling pathways, we conducted western blot assays. The results revealed that overexpressing the Flag-Metrnl plasmid in RAW264.7 cells inhibited NF-κB translocation from the cytoplasm to the nucleus and increased the expression of IκBα in the cytoplasm (**Figure 6A**). This phenomenon could be reversed by the overexpression of UCHL-1. Similarly, when Metrnl was inhibited in RAW264.7 cells, cytoplasmic IκBα and NF-κB were down-regulated, while nuclear NF-κB was up-regulated. Simultaneous knockdown of UCHL-1 could reverse this condition (**Figure 6B**). Moreover, the above results were verified in THP-1 cells (**Figures 6C, D**). To further examine the biological process, supernatants were collected from macrophages with knocked down Metrnl or concurrently knocked down Metrnl and UCHL-1. We treated HUVECs with these supernatants and performed tube formation assays (**Figures 6E, F**). The results illustrated that the deficiency of Metrnl affected the biological function of endothelial cells induced by the inflammation of macrophages. Taken together, our findings suggest that Metrnl inhibited the angiogenic capabilities of HUVECs by interacting with UCHL-1 in macrophages, which was linked to the NF-κB signaling pathway.

## Discussion

Herein, we found that Metrnl could stabilize endothelial function and inhibit angiogenesis in a CNV model by mitigating the inflammatory microenvironment. Mechanistic studies revealed that Metrnl interacts with UCHL-1 and regulates the NF-κB signaling pathway, thereby inhibiting the secretion of inflammatory factors in macrophages. The data support the potential therapeutic opportunities of Metrnl to prevent the progression of CNV.

AMD is one of the most common irreversible blinding eye diseases in the elderly, resulting from a complex combination of metabolism, genetics, and the environment (14). Currently, anti-VEGF therapy is the main treatment for neovascular AMD (15, 16). However, there is no significant response effect in some patients (17). VEGF receptor activation induces macrophages to produce pro-inflammatory and pro-angiogenic mediators (18). Therefore, single-targeted anti-VEGF therapeutic regimens are not effective in inhibiting angiogenesis (19). CNV is a crucial feature of the late stage of wet AMD. Recent studies have suggested that controlling inflammatory factors is a positive strategy for inhibiting the progress of CNV. Consistent with other reports (20, 21), the expressions of inflammatory factors were validated to increase in the aqueous humor of wAMD patients and choroid of CNV mice. It is worth noting that the expression of Metrnl was significantly decreased in the choroid-sclera complex of CNV mice. However, in the aqueous humor of wAMD patients, although the expression of Metrnl was decreased, the difference was not statistically significant, which may be attributed to the inability of aqueous humor to directly reflect the change of cytokines in the posterior segment of the eye and the small number of subjects in this research.

It is well established that the number of macrophages is remarkably increased in wAMD patients (22) and is associated with inflammation (23). A study demonstrated that it is a feasible method to control the activation of macrophages to inhibit CNV. Metrnl was validated to weaken inflammatory responses in LPS-treated macrophages by downregulating pro-inflammatory factors such as IL-6 and TNF-α (24). In this study, the overexpression of Metrnl in macrophages could resist LPS-induced inflammatory responses. Exogenous Metrnl also restricted choroidal inflammatory responses in CNV mice. Activated macrophages secrete cytokines that have angiogenic and pro-inflammatory activity to allow endothelial cell migration and form new capillaries (25). Our findings showed that Metrnl ameliorated the LPS-induced inflammatory microenvironment in macrophages, thereby inhibiting angiogenesis *in vitro* and *in vivo*. However, in a myocardial infarction model, Metrnl demonstrated angiogenic effects through KIT-dependent signaling pathways (26). Metrnl deficiency in endothelial cells influences the migration and tube formation ability of endothelial cells, delaying wound healing (27). This discrepancy may be attributed to the different models. In our study, Metrnl regulated inflammation in macrophages to affect the function of endothelial cells. Furthermore, tight junctions between endothelial cells are essential for maintaining vascular permeability (28). Vascular endothelial cadherin (VE-cadherin) is a key component of intercellular connections (28, 29). Inflammation has been demonstrated to impact these connections between endothelial cells, leading to impaired vascular function (30). Recent experimental evidence highlights that the deficiency of Metrnl results in endothelial cell dysfunction (31). Moreover, our observations revealed that the overexpression of Metrnl in macrophages plays a protective role in maintaining the integrity of endothelial cell-cell junctions. These findings collectively validate that Metrnl exerts anti-inflammatory effects in the CNV process through its influence on macrophages.

Previous studies have emphasized Metrnl could regulate inflammation through various mechanisms, such as attenuating lipid-induced inflammation and LPS-mediated endothelial cell inflammation via AMPK or PPARδ-dependent pathways (24, 32). In the present study, employing protein mass spectrometry and protein molecular docking techniques, we identified that Metrnl binds to UCHL-1 to modulate the NF-kB signaling pathway, thereby influencing the regulation of macrophage inflammatory factors. UCHL-1, categorized as a deubiquitinase (DUB), plays a role in removing attached ubiquitin from substrates, preventing protein degradation by proteasomes, and disrupting signal transmission via ubiquitin (33). Elevated UCHL-1 levels have been associated with inflammatory diseases, and its inhibitor has been shown to reduce inflammatory cell infiltration (34, 35). In addition, UCHL-1 has been implicated in promoting inflammation through the NF-κB pathways in LPS-associated macrophages (36). Notably, Metrnl has been shown to down-regulate NF-κB pathways to inhibit inflammation (24). To demonstrate Metrnl’s dependence on UCHL-1 in regulating NF-κB pathways, we manipulated the expression of Metrnl and observed changes in NF-κB pathways. Our results indicated that increased Metrnl expression in LPS-induced macrophages inhibited NF-κB translocation from the cytoplasm to the nucleus. However, this effect was attenuated when UCHL-1 levels were up-regulated. Therefore, we propose that Metrnl interacts with UCHL-1 to inhibit deubiquitination activity, allowing the continuous ubiquitination degradation of NF-κB and consequently weakening the inflammatory response.

Overall, our findings demonstrate that Metrnl can effectively inhibit choroidal neovascularization by mitigating the inflammatory microenvironment through the UCHL-1/NF-κB signaling pathway in macrophages. Metrnl, as an anti-inflammatory factor, inhibits neovascularization by ameliorating the persistent inflammatory environment of the choroid. It suggests that Metrnl holds promise as a potential therapeutic agent for combating CNV.

## Data availability statement

The raw data supporting the conclusions of this article will be made available by the authors, without undue reservation.

## Ethics statement

The studies involving humans were approved by the institute’s ethics committee at Chongqing University Jiangjin Hospital (approval reference number: KY2023006). The studies were conducted in accordance with the local legislation and institutional requirements. The participants provided their written informed consent to participate in this study. The animal study was approved by the Experimental Animal Welfare Ethics Committee of Chongqing University of Arts and Sciences (approval reference number: CQWLDF202304). The study was conducted in accordance with the local legislation and institutional requirements.

## Author contributions

LZ: Conceptualization, Investigation, Methodology, Writing – original draft. YL: Formal analysis, Writing – original draft. ZW: Investigation, Methodology, Writing – original draft. QS: Software, Writing – review & editing. CZ: Methodology, Writing – review & editing. HL: Data curation, Writing – review & editing. XZ: Funding acquisition, Writing – review & editing. JY: Investigation, Writing – original draft. QL: Investigation, Writing – original draft. DT: Supervision, Writing – review & editing. KO: Project administration, Validation, Writing – review & editing. YF: Funding acquisition, Supervision, Writing – review & editing.

## Supplementary material

Supplementary Figure 1 The cell viability and proliferation of endothelial cells and macrophages were not affected by Metrnl stimulation when the dosage was within a certain range. **(A–C)** The cell viability of HUVECs was detected by CCK-8 and EdU assays. The data showed the cell viability was not affected by Metrnl when the dosage did not exceed 200 ng/ml. **(D, E)** The cell viability of RAW264.7 cells was detected by EdU. Data are represented as mean ± SEM, from 3 independent experiments, scale bar, 100 μm. (ns: p > 0.05).

Supplementary Figure 2 The interaction of Metrnl with other proteins. **(A, B)** Western blot showed that BDB did not bind OTUD6B or CPT1B in HEK293 cells.

Supplementary Figure 3 Laser induced mouse CNV model. **(A)** Fundus photography showed successful CNV modeling. **(B)** Immunohistochemical analysis of tissue samples from mice.

Supplementary Figure 4 Plasmid profile. The detailed information about plasmid was displayed in the graph.

Supplementary Figure 5
**(A)** A part of the results about protein binding mass spectrometry. **(B)** mRNA levels of VEGF in RAW264.7 cells. Data are expressed as means ± SD, from 3 independent experiments. (ns >.05).

Supplementary Table 1 The primer sequences were designed from the PrimerBank database.
