# Supplementary figures and images for: Metrnl inhibits choroidal neovascularization by attenuating the choroidal inflammation via inactivating the UCHL-1/NF-κB signaling pathway
Source: Front Immunol. 2024 Apr 30;15:1379586. doi: 10.3389/fimmu.2024.1379586 (PMC11091344; doi:10.3389/fimmu.2024.1379586)

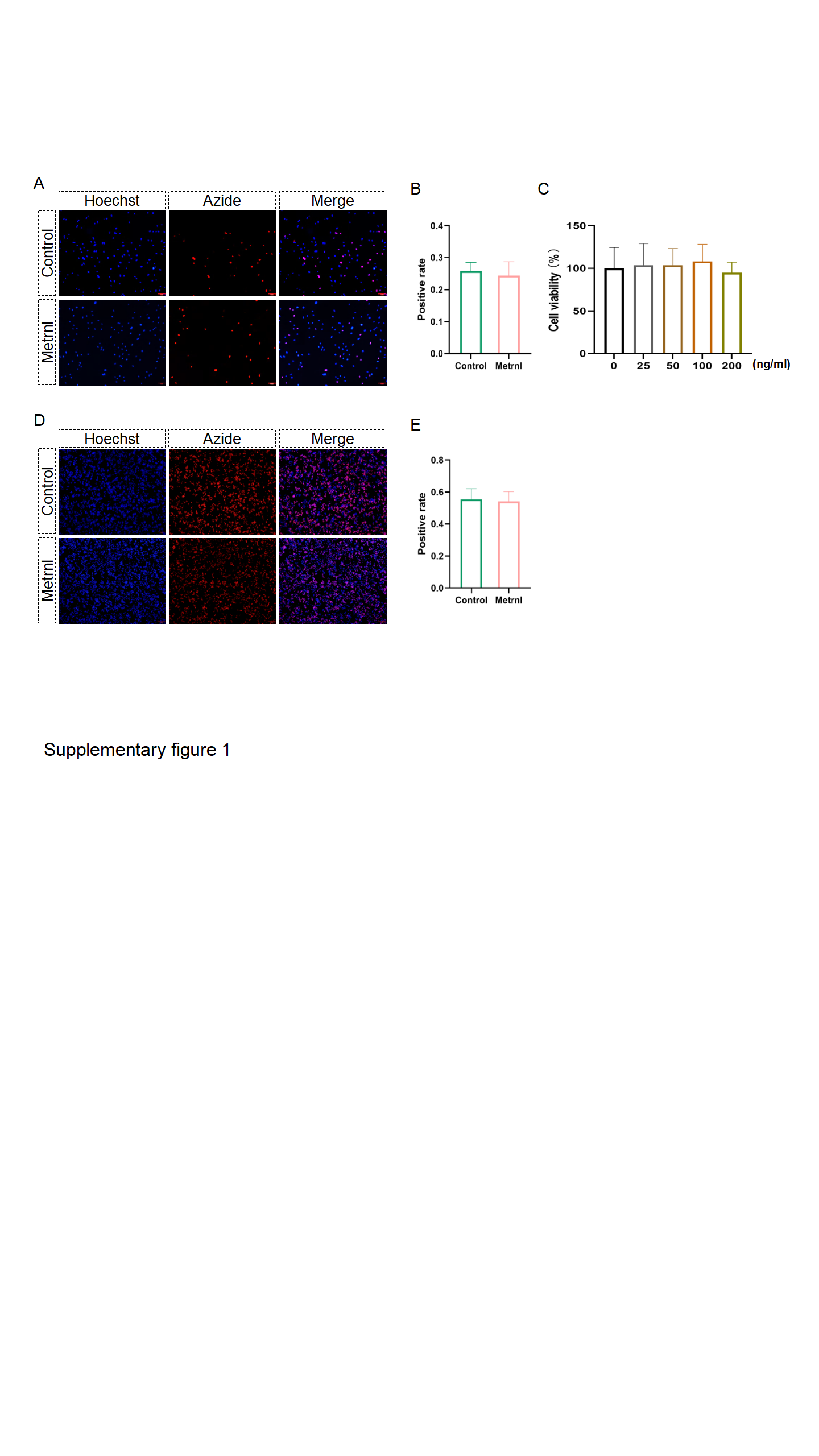

Supplement: Supplementary Figure 1 — The cell viability and proliferation of endothelial cells and macrophages were not affected by Metrnl stimulation when the dosage was within a certain range. (A–C) The cell viability of HUVECs was detected by CCK-8 and EdU assays. The data showed the cell viability was not affected by Metrnl when the dosage did not exceed 200 ng/ml. (D, E) The cell viability of RAW264.7 cells was detected by EdU. Data are represented as mean ± SEM, from 3 independent experiments, scale bar, 100 μm. (ns: p > 0.05). [file DataSheet_1.zip › Supplementary Figure 1.TIF]

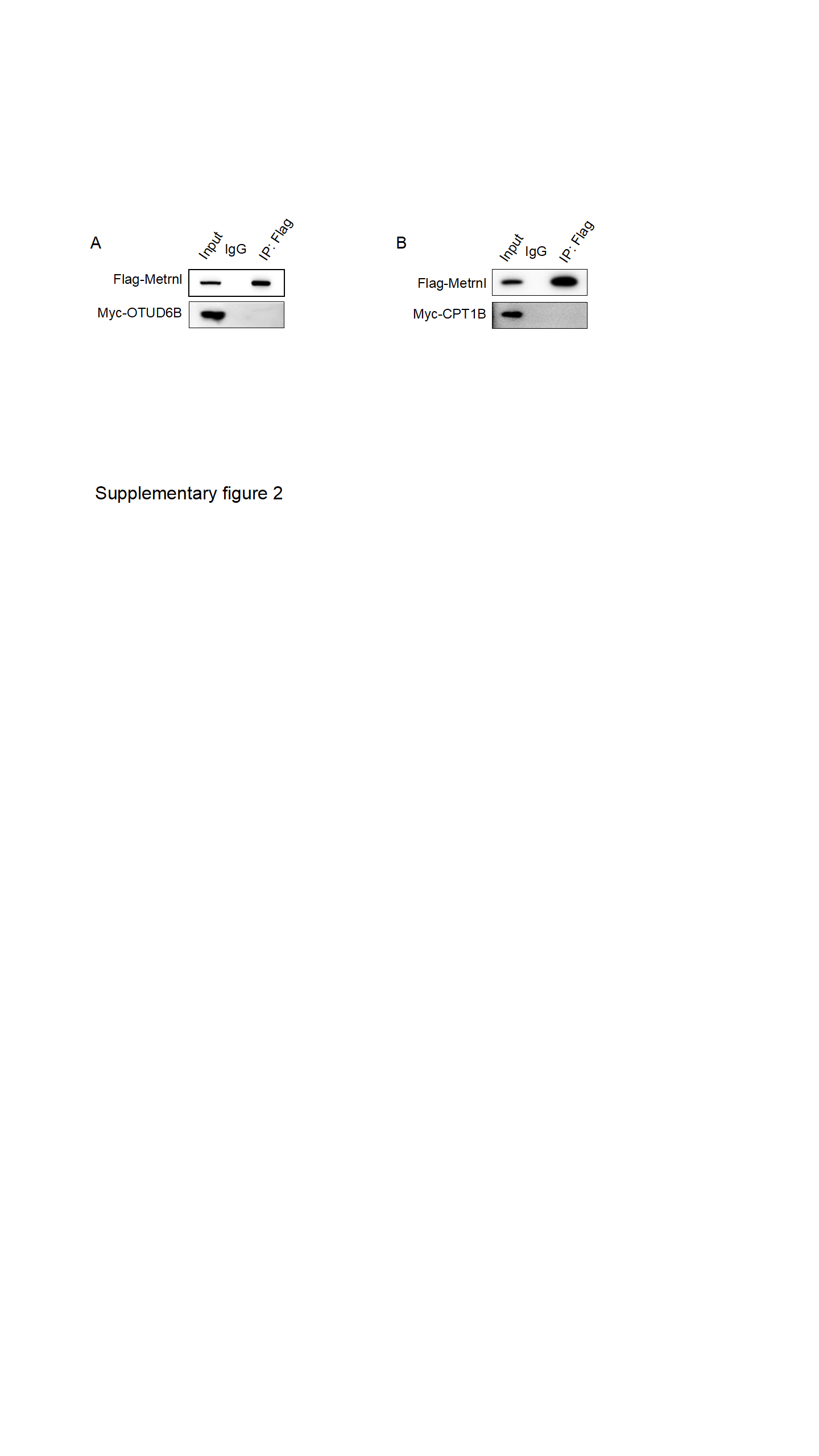

Supplement: Supplementary Figure 1 — The cell viability and proliferation of endothelial cells and macrophages were not affected by Metrnl stimulation when the dosage was within a certain range. (A–C) The cell viability of HUVECs was detected by CCK-8 and EdU assays. The data showed the cell viability was not affected by Metrnl when the dosage did not exceed 200 ng/ml. (D, E) The cell viability of RAW264.7 cells was detected by EdU. Data are represented as mean ± SEM, from 3 independent experiments, scale bar, 100 μm. (ns: p > 0.05). [file DataSheet_1.zip › Supplementary Figure 2.TIF]

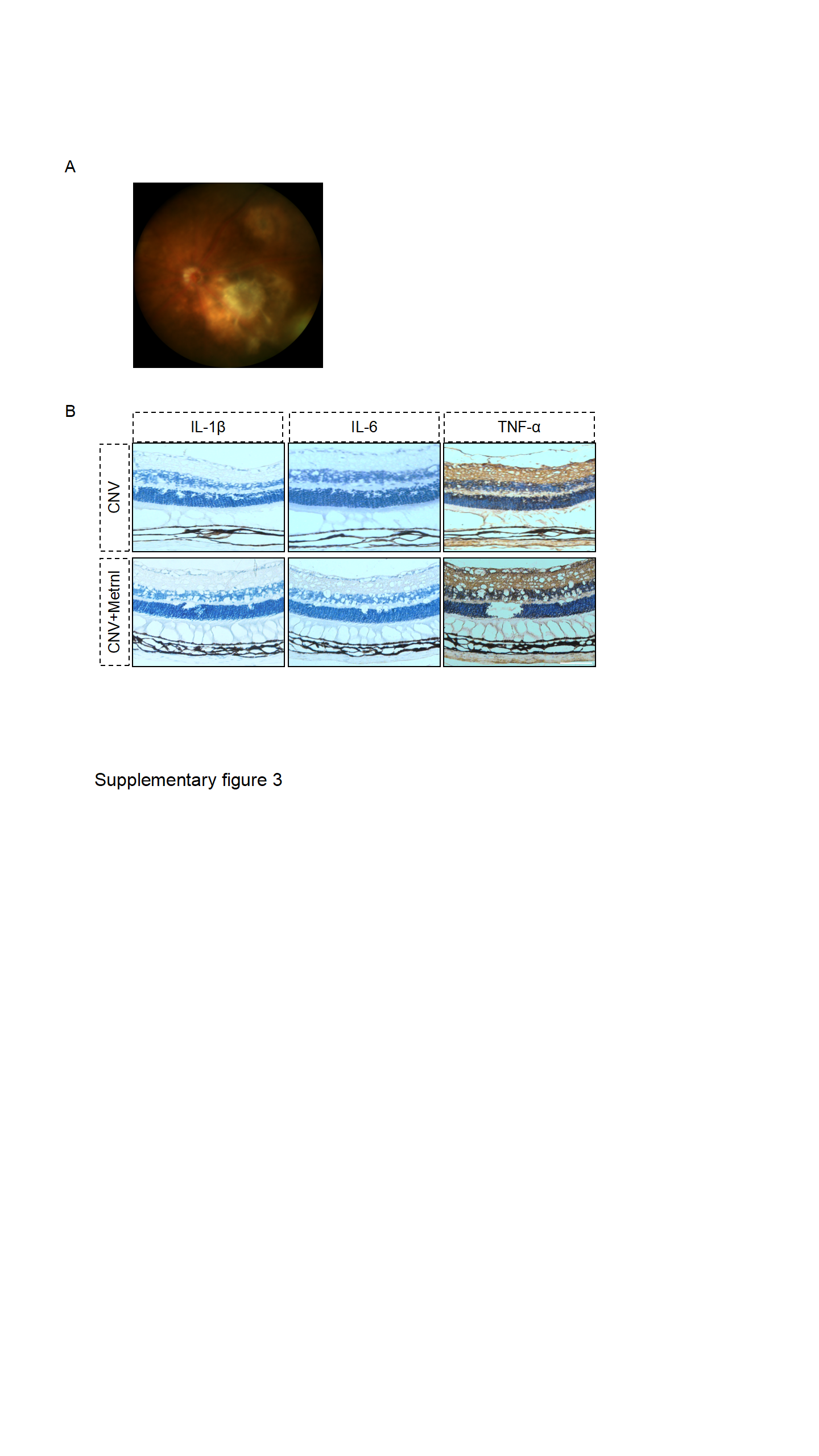

Supplement: Supplementary Figure 1 — The cell viability and proliferation of endothelial cells and macrophages were not affected by Metrnl stimulation when the dosage was within a certain range. (A–C) The cell viability of HUVECs was detected by CCK-8 and EdU assays. The data showed the cell viability was not affected by Metrnl when the dosage did not exceed 200 ng/ml. (D, E) The cell viability of RAW264.7 cells was detected by EdU. Data are represented as mean ± SEM, from 3 independent experiments, scale bar, 100 μm. (ns: p > 0.05). [file DataSheet_1.zip › Supplementary Figure 3.TIF]

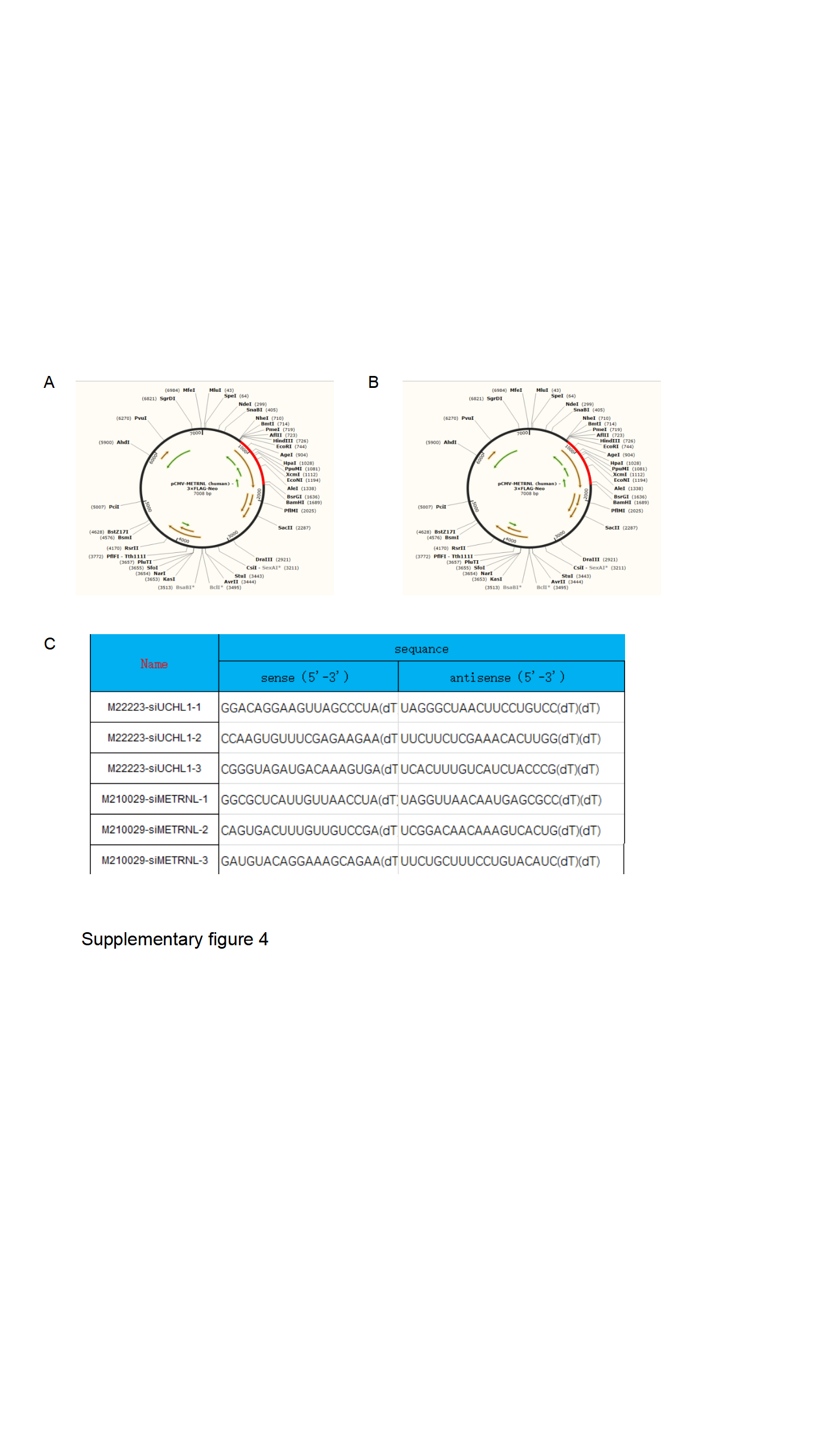

Supplement: Supplementary Figure 1 — The cell viability and proliferation of endothelial cells and macrophages were not affected by Metrnl stimulation when the dosage was within a certain range. (A–C) The cell viability of HUVECs was detected by CCK-8 and EdU assays. The data showed the cell viability was not affected by Metrnl when the dosage did not exceed 200 ng/ml. (D, E) The cell viability of RAW264.7 cells was detected by EdU. Data are represented as mean ± SEM, from 3 independent experiments, scale bar, 100 μm. (ns: p > 0.05). [file DataSheet_1.zip › Supplementary Figure 4.TIF]

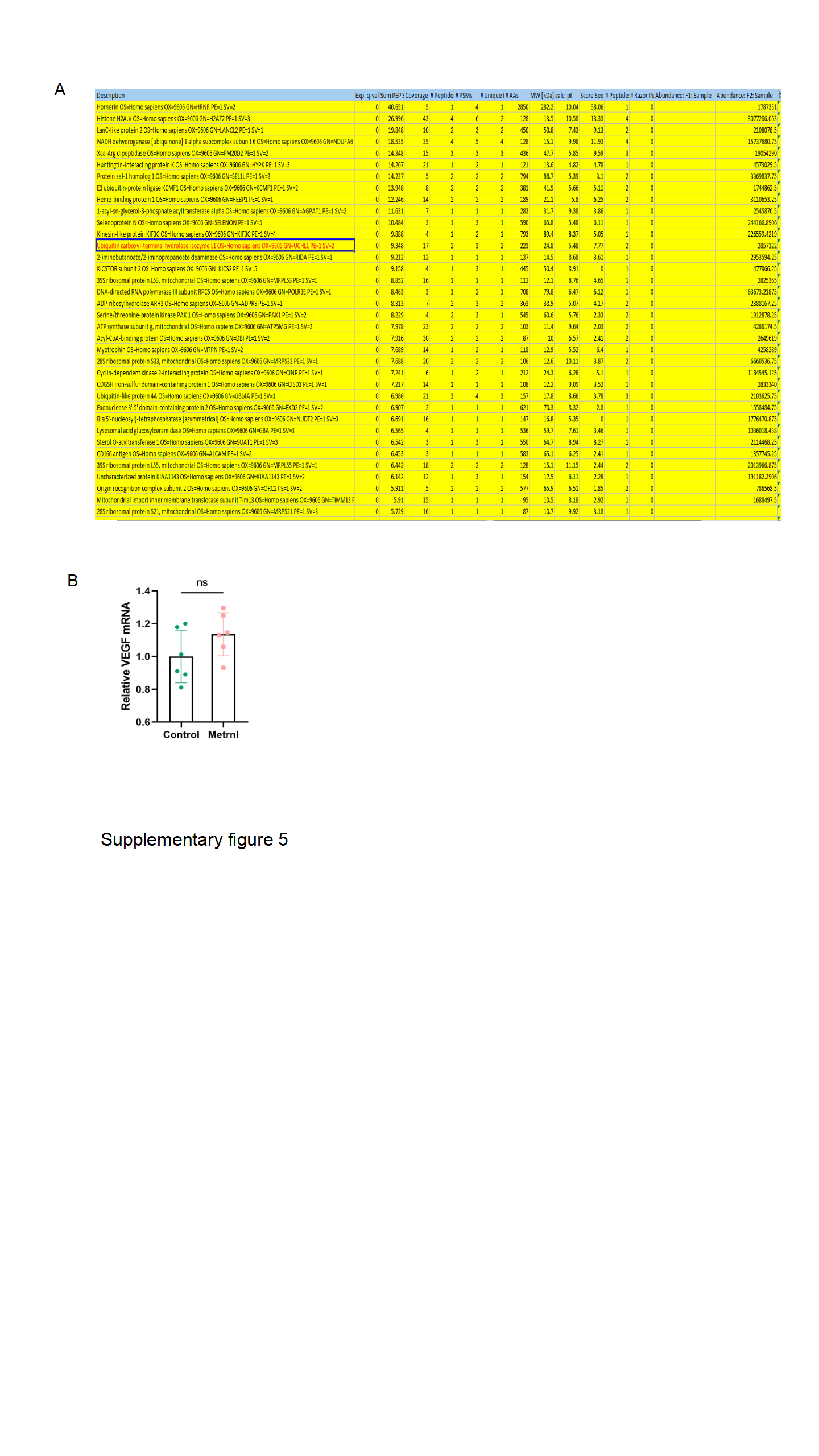

Supplement: Supplementary Figure 1 — The cell viability and proliferation of endothelial cells and macrophages were not affected by Metrnl stimulation when the dosage was within a certain range. (A–C) The cell viability of HUVECs was detected by CCK-8 and EdU assays. The data showed the cell viability was not affected by Metrnl when the dosage did not exceed 200 ng/ml. (D, E) The cell viability of RAW264.7 cells was detected by EdU. Data are represented as mean ± SEM, from 3 independent experiments, scale bar, 100 μm. (ns: p > 0.05). [file DataSheet_1.zip › Supplementary Figure 5.TIF]

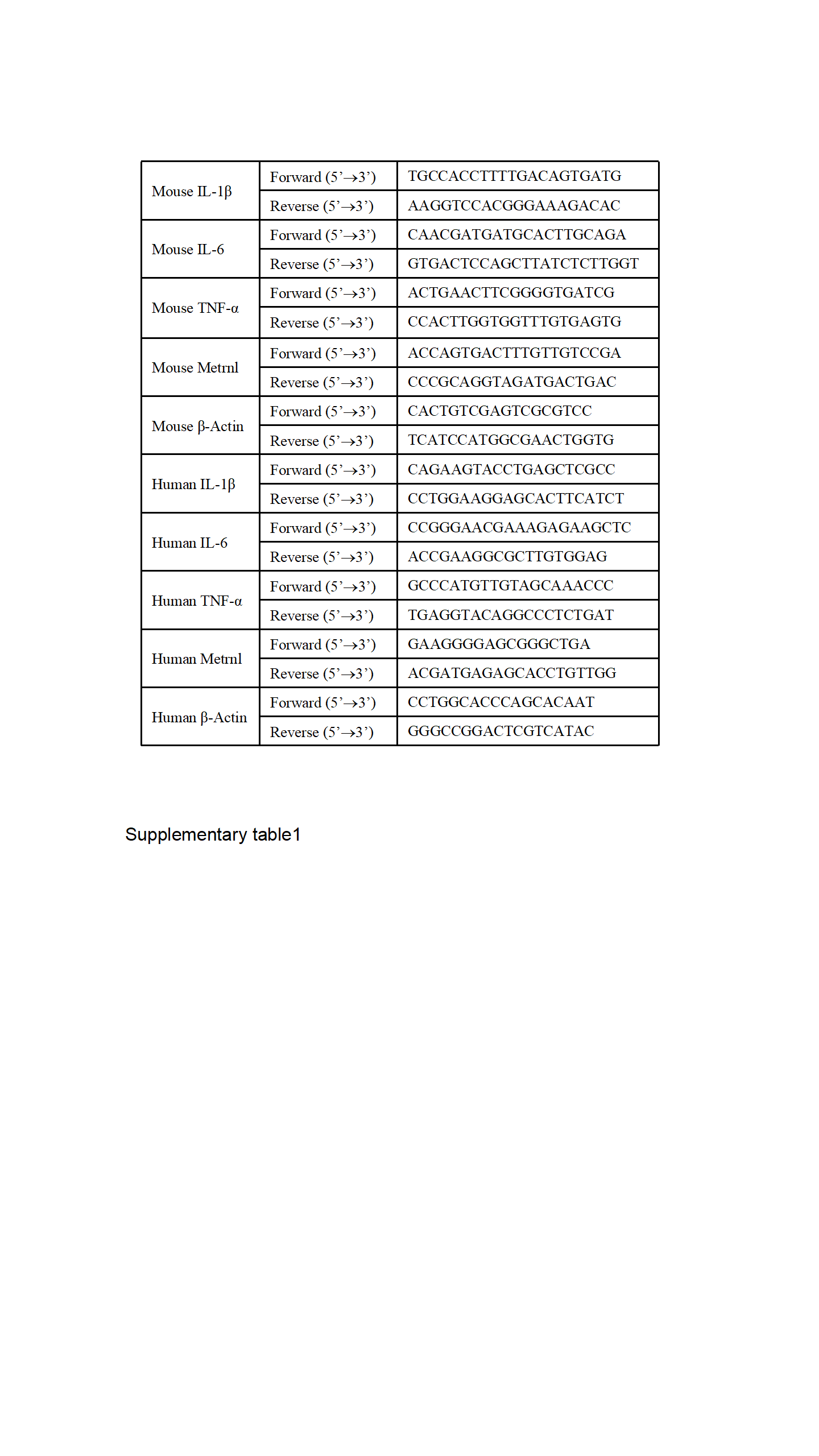

Supplement: Supplementary Figure 1 — The cell viability and proliferation of endothelial cells and macrophages were not affected by Metrnl stimulation when the dosage was within a certain range. (A–C) The cell viability of HUVECs was detected by CCK-8 and EdU assays. The data showed the cell viability was not affected by Metrnl when the dosage did not exceed 200 ng/ml. (D, E) The cell viability of RAW264.7 cells was detected by EdU. Data are represented as mean ± SEM, from 3 independent experiments, scale bar, 100 μm. (ns: p > 0.05). [file DataSheet_1.zip › Supplementary Table 1.TIF]
